# How can we accurately measure disease activity during pregnancy in systemic lupus erythematosus? New insights from the BILAG-2004 Pregnancy Index

**DOI:** 10.1093/rap/rkad024

**Published:** 2023-02-16

**Authors:** Sasha Ali, Carmen Garcia, Serena Fasano, Charles Raine, Chris Wincup

**Affiliations:** Department of Rheumatology, King’s College Hospital, London, UK; Department of Rheumatology, King’s College London, London, UK; Department of Rheumatology, King’s College London, London, UK; Department of Rheumatology, University Hospital of Basurto, Bilbao, Spain; Rheumatology Unit, Department of Precision Medicine, University of Campania, “Luigi Vanvitelli”, Naples, Italy; Department of Rheumatology, Division of Medicine, University College London, London, UK; Department of Rheumatology, King’s College Hospital, London, UK; Department of Rheumatology, Division of Medicine, University College London, London, UK

**This editorial refers to ‘The BILAG2004-Pregnancy Index is a valid disease activity outcome measure for pregnant SLE patients’, by Yee *et al.*https://doi.org/10.1093/rap/rkac081.**

Pregnancy is a common consideration for clinicians managing patients with SLE, a disease that predominantly affects women of childbearing age. This scenario presents several challenges, in particular when determining whether symptoms are related to increased disease activity, attributable to the physiological state of pregnancy or are other non-SLE pregnancy-related symptoms that might mimic the disease. Adequate control of SLE in pregnancy is vital, because active disease is associated with an increased risk of a range of adverse pregnancy outcomes (APOs) [1, 2]. It has also been noted that APO increases the risk of subsequently developing autoimmune rheumatic diseases (such as SLE), further demonstrating the complex immunological changes that occur during pregnancy [3].

It is paramount that treatment decisions in pregnancy rely on tools that provide accurate disease activity assessment. In the manuscript by Yee *et al.* [4] presented in this edition of the journal, the authors sought to assess whether the BILAG2004-Pregnancy Index (BILAG2004-P), a modification of the commonly used BILAG-2004 Index, could sensitively detect changes in SLE disease activity across the duration of pregnancy over time.

Quantification of lupus disease activity in pregnancy remains an area of extensive debate, with the reported incidence of flares and APOs varying widely across numerous studies in the literature. It has been suggested that disease flares occur more frequently during pregnancy in lupus when compared with other autoimmune rheumatic diseases [5]. A 2015 study by Buyon *et al.* [6] noted that severe flares were observed in 3% of lupus pregnancies with stable mild or moderate disease at the time of conception (however, patients with severe disease at conception were excluded from the study). In a retrospective multicentre study, Radin *et al.* [7] reported that disease activity at conception also predicted subsequent risk of flare for ≤2 years following birth, with remission at the time of becoming pregnant conferring significantly better outcomes across gestation, the peripartum period and beyond. The importance of accurately assessing disease activity during pregnancy in SLE has also been demonstrated in a study by Harris *et al.* [8], in which both patient and physician assessment of disease activity in pregnancy was assessed as predictors of future APOs (including lupus flare). In pregnant women with RA, both clinician and patient evaluation of disease activity were correlated closely with pregnancy outcomes; however, in SLE only the physician (but not the patient) assessment was associated with adverse outcomes. It is therefore vital for physicians to have a robust measure of lupus disease activity, in order to provide women with reliable information relating to risks of disease flare and APOs in pregnancy. Measurements of SLE disease activity during pregnancy should be both accurate and reproducible, while also sensitively differentiating symptoms relating to active SLE from similar, but non-lupus-related symptoms that can also occur during pregnancy, such as non-specific musculoskeletal pain and fatigue. A lack of validity and poor sensitivity to change over the course of pregnancy are major limitations for potential instruments used to monitor SLE activity in both clinical practice and research studies.

The BILAG-2004 index is a systems-based disease activity measure that has been extensively validated for use in both clinical practice and research outside of the setting of pregnancy [9]. In 2012, the BILAG-2004 index was updated for use when measuring lupus disease activity in pregnancy [10]. The BILAG-P version of the activity score maintained the nine core domains of the standard BILAG-2004 index and specifically accounted for the physiological changes seen in pregnancy that might result in confusion when appraising symptoms potentially related to lupus in clinical practice. Previously, it has been reported that disease activity within certain organ domains confers greater risk of APO (namely renal, haematological and serositis) [2], which might be detected sensitively by the individual organ-based component assessment used in the BILAG index. Thus, the BILAG-P was effective for use when applied to the evaluation of SLE activity in pregnancy.

The present study by Yee *et al.* [4] assesses whether the BILAG-P is both valid and sensitive to change throughout pregnancy in a group of 97 patients (with a total of 112 pregnancies) recruited from five specialist lupus centres in England. The authors conducted an observational cross-sectional study to test whether BILAG-P had both construct validity (measured through standard laboratory measures of SLE disease activity, including complement C3 and C4 levels and anti-dsDNA antibody titres) and criterion validity (using the physician global assessment score) over the course of pregnancy. The authors defined sensitivity to change by identifying alterations in treatment between two successive visits. Longitudinal disease activity assessment in pregnancy is of particular importance, especially in those patients at higher risk of APOs. It was found that an increase in BILAG-P score was associated with serological disease activity (low complement C3), a corresponding increase in therapy and a higher physician global assessment score, thus representing a robust measure for detecting changes in disease activity throughout pregnancy. It should be noted that there was a low prevalence of patients with active disease (15.7% of assessments in the study had a BILAG-P grade A or B). However, this should not affect external validity of the study, because it would be expected that the standard of care in SLE patients would be to ensure that disease activity is low before embarking upon pregnancy.

A potential limitation of BILAG-P is that it can be more time consuming than other disease activity measures. Furthermore, a level of familiarity and proficiency with the BILAG index might be required. Training in BILAG-P scoring is likely to be required, particularly for use in the research setting. However, with the advent of the new Easy-BILAG [11], this instrument should become more widely used in clinical practice.

In conclusion, this study demonstrates that the BILAG-P is a useful tool to measure lupus disease activity throughout the course of pregnancy in both the clinical and research settings. Increased incorporation of the BILAG-P into clinical practice is likely to benefit future patient care through accurate stratification of treatment decisions and improved pregnancy outcomes. Ultimately, this will increase the confidence of both lupus patients and their physicians when managing the disease throughout pregnancy.

## Data Availability

There are no original data presented in this Editorial. Therefore, data are not available.
