# Correction to: How can we accurately measure disease activity during pregnancy in systemic lupus erythematosus? New insights from the BILAG-2004 Pregnancy Index

**DOI:** 10.1093/rap/rkad043

**Published:** 2023-04-27

This is a correction to: Sasha Ali, Carmen Garcia, Serena Fasano, Charles Raine, Chris Wincup, How can we accurately measure disease activity during pregnancy in systemic lupus erythematosus? New insights from the BILAG-2004 Pregnancy Index, *Rheumatology Advances in Practice*, Volume 7, Issue 1, 2023, rkad024, https://doi.org/10.1093/rap/rkad024

Upon original publication, author Charles Raine’s name was inadvertently omitted from the author list in the PDF version of the manuscript.

The Publisher apologizes for this error, which has now been corrected.

